# First person – Louise Roberts

**DOI:** 10.1242/bio.045880

**Published:** 2019-07-15

## Abstract

First Person is a series of interviews with the first authors of a selection of papers published in Biology Open, helping early-career researchers promote themselves alongside their papers. Louise Roberts is first author on ‘Finding a home in the noise: cross-modal impact of anthropogenic vibration on animal search behaviour’, published in BIO. Louise is a Postdoctoral Research Associate in the lab of Mark Laidre at Dartmouth College, USA, investigating how animals interpret their environment using sound in the air and the water, and vibration within sediments and surfaces.


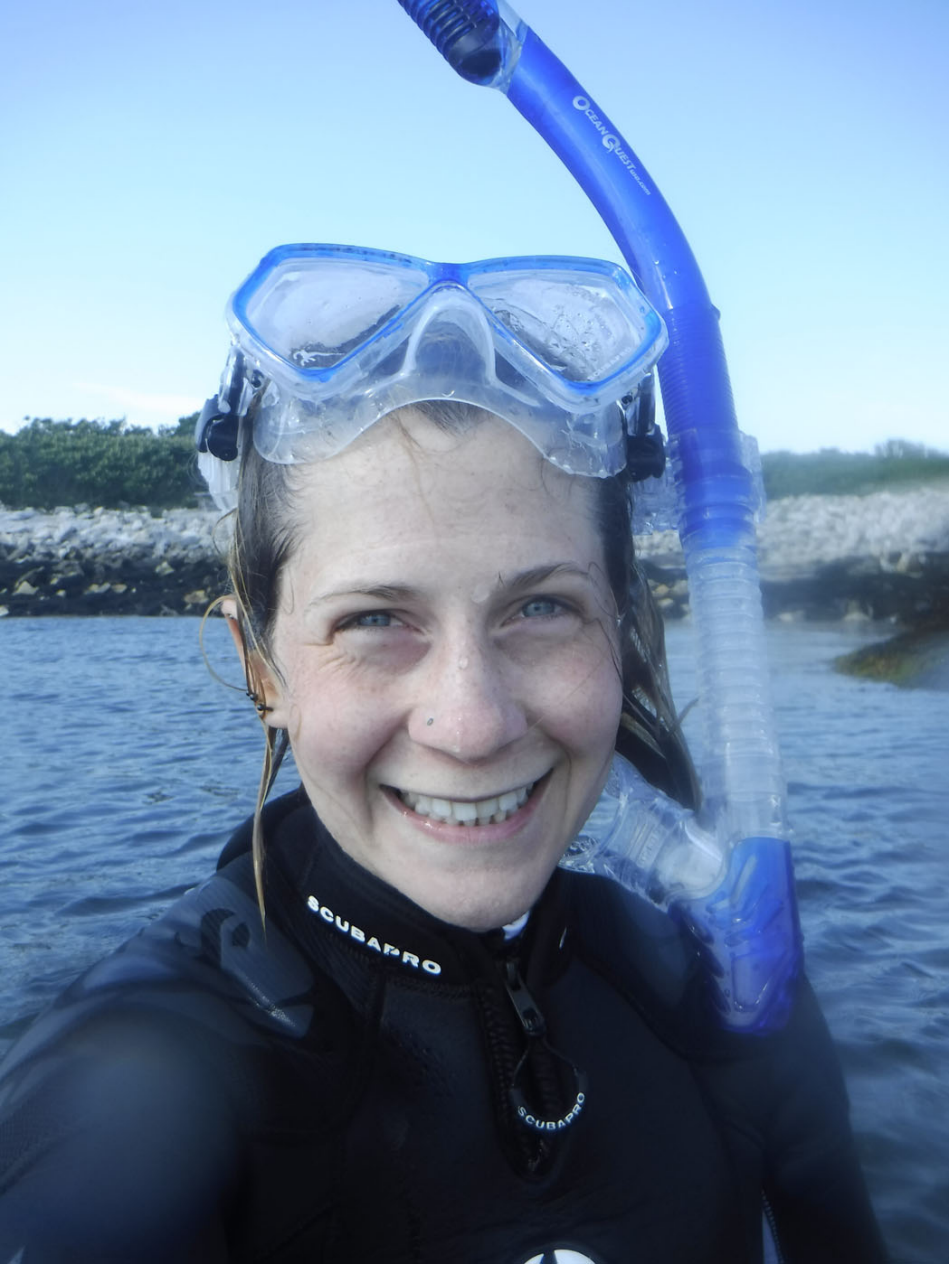


**Louise Roberts**

**What is your scientific background and the general focus of your lab?**

I am a bioacoustician and a budding biotremologist interested in the behaviour of animals. My background is in marine biology, and I have always had a strong interest in crustaceans in particular. After an undergraduate degree at Durham University (UK) and a master's degree at Bangor University (UK), I moved on to the University of Hull (UK) for my doctorate. It was there, within the Institute of Estuarine and Coastal Studies, that I became passionate about understanding the impact of humans upon the marine environment, and about bioacoustics. My doctorate focused on the effects of anthropogenic noise on the behaviour of fish and invertebrates, an area I remained active within in subsequent postdoctoral research in the UK. I moved across the pond a couple of years ago to Dartmouth College (USA) to become a postdoctoral research associate in the Laidre laboratory, a lab currently focused on the behaviour, sensory and evolutionary ecology of hermit crabs. Here, I explored the vibroacoustic behaviour of terrestrial hermit crabs with field seasons based in Costa Rica. To keep one foot in the ocean during that time, I applied for an adjunct Scientist-in-Residence Fellowship at Shoals Marine Laboratory (a University of New Hampshire and Cornell facility). As part of the fellowship I lived on Appledore Island off the coast of Maine for a summer, which allowed me to return to my underwater noise interests and carry out the experiments in this paper.

**How would you explain the main findings of your paper to non-scientific family and friends?**

Chemical cues are important for marine organisms, which use them to find key resources and sense their surroundings. Hermit crabs use empty gastropod (snail) shells as their ‘homes’, residing inside them for protection. They are constantly on the hunt for these new shells, which they find using chemical cues. We found that underwater noise impacted the search behaviour of hermit crabs, with fewer crabs attracted to a chemical cue (representative of new home) after noise exposure. So, noise appeared to affect the hermit crabs' search for a new home.

**What are the potential implications of these results for your field of research?**

This work is particularly exciting since it is one of only a few indicating that noise affects behaviour cross-modally. That is, a vibroacoustic cue (a sound or a vibration in a substrate) is impacting a behaviour mediated by a chemical cue, so the effect crosses between two sensory modes. Additionally, there are few studies exposing free-ranging benthic invertebrates to anthropogenic noise, since the logistics of such experiments can be demanding! Yet the behaviour of many animals changes in captivity and acoustics within small laboratory tanks are complex, so field experiments are valuable. I will be presenting this research at the 2019 Effects of Noise on Aquatic Life Conference in Den Haag, the Netherlands, this summer and I'm looking forward to discussing the results with my peers!

“There are few studies exposing free-ranging benthic invertebrates to anthropogenic noise, since the logistics of such experiments can be demanding!”

**What has surprised you the most while conducting your research?**

From my previous research we knew that marine hermit crabs were sensitive to noise and sediment vibration. However we were still surprised that the rather low amplitude of our manual stimulus (which was just me, hammering in the subtidal) could have such an effect upon the hermit crabs’ behaviour!

**Figure:**
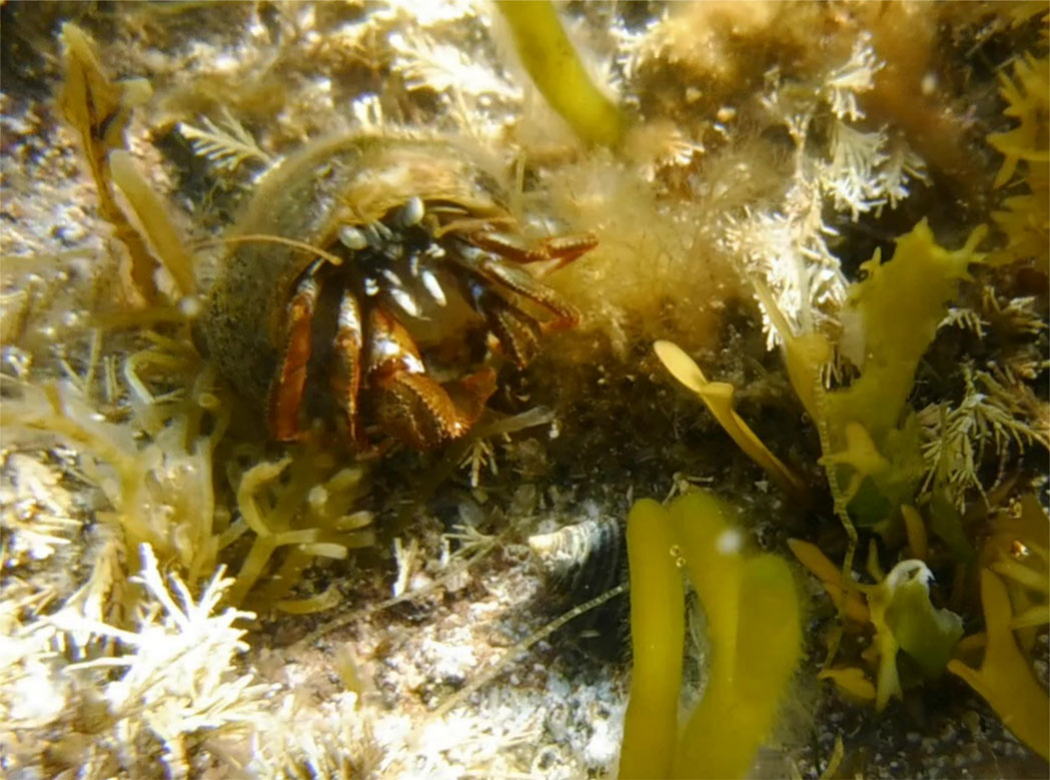
***Pagurus acadianus,* the study species exposed to anthropogenic noise in this paper. Photo credit: L. Roberts.**

**What changes do you think could improve the professional lives of early-career scientists?**

Postdocs are fundamental to research laboratories, yet are often undervalued and mistreated. As such, institutions should re-evaluate how they support postdocs, and implement better systems to ensure that the postdoc/principal-investigator working relationship is healthy. I encourage postdocs in the US to look at the supportive resources of the National Postdoctoral Association, and also to look out for their fellow postdocs so that no one suffers in silence.

**What advice would you give to other early-career researchers?**

I try and always say ‘yes’ to academic opportunities. I used to have a dislike for public speaking, but after many years of always saying ‘yes’ when asked to give a presentation, I now really enjoy giving them! I'd also advise early-career researchers to be flexible about where and what they work on, to make the most of opportunities that they encounter.

“Always say ‘yes’ to academic opportunities.”

## References

[BIO045880C1] Roberts L. and Laidre M. L. (2019). Finding a home in the noise: cross-modal impact of anthropogenic vibration on animal search behaviour. *Biol. Open* 8, 041988 10.1242/bio.041988 PMC6679394 31292133

